# Atomically Surficial Modulation in Two-Dimensional Semiconductor Nanocrystals for Selective Photocatalytic Reactions

**DOI:** 10.3389/fchem.2022.890287

**Published:** 2022-04-14

**Authors:** Shuwen Zhu, Xinyuan Li, Jiatao Zhang

**Affiliations:** MOE Key Laboratory of Cluster Science, School of Chemistry and Chemical Engineering, Beijing Institute of Technology, Beijing, China

**Keywords:** semiconductor nanocrystals, two-dimensional photocatalysts, surficial modulation, surface vacancies, single-atom modification, dual-site components

## Abstract

Photocatalysis, directly converting solar energy into chemical energy, is identified as an ideal strategy to reduce the increasing consumption of fossil fuels and facilitate carbon neutralization. In the past few years, a great number of endeavors have been devoted to developing photocatalysts with a high conversion efficiency and selectivity. Atomically surficial modulation strategies, including surface vacancies, single-atom modification, and dual-site components, exhibited positive impacts on tuning key steps of photocatalytic reactions. In this mini-review, we focus on the latest progress of the atomically surficial modulations on two-dimensional semiconductor photocatalysts and their role in enhancing selectively photocatalytic performance. We hope that this mini-review could provide new insights for researchers on nanosynthesis and photocatalysis.

## Introduction

**FIGURE 1:**
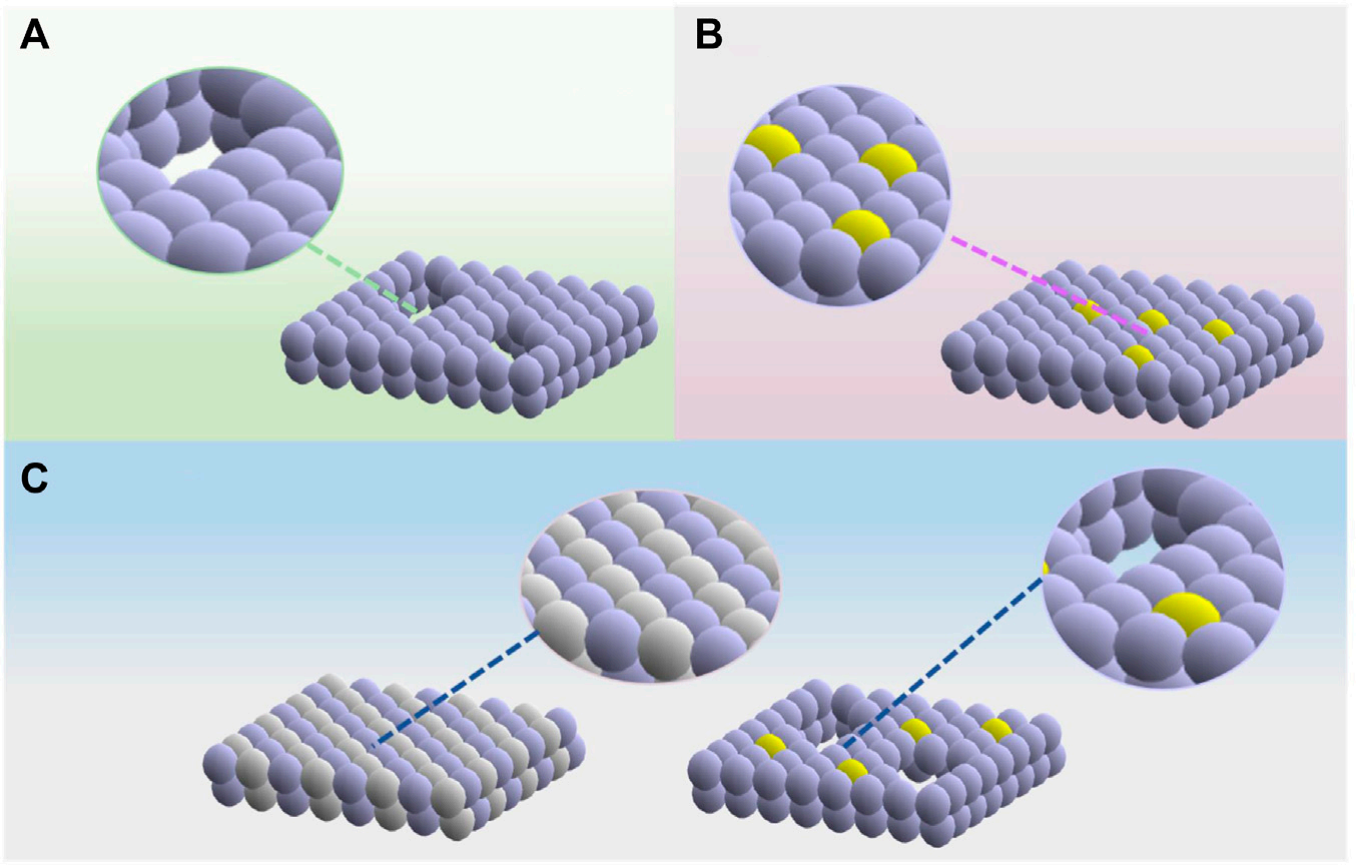
Surficial modulations on 2D semiconductor photocatalysts discussed in this mini-review. **(A)** Surface vacancies. **(B)** Single-atom modification. **(C)** Dual-site components.

Photocatalysis is regarded as an efficient strategy to directly convert solar energy into chemical energy, which provides a solution to release the increasingly serious shortage of natural resources (Bai et al., 2017; Gao et al., 2020). Photocatalytic reactions are mainly driven by photo-induced charge carriers, and the reaction process could be divided into charge generation, separation, migration, and surficial reactions (Li et al., 2016; Gao et al., 2017; Yang et al., 2021). The utilization efficiency of carriers in each step determines the overall performance of photocatalysis (Li A et al., 2019). The two-dimensional (2D) nanocrystals with thin thicknesses are attractive candidates for photocatalysts by virtue of the short carrier transport distance and thus allow for improved charge separation (Tan et al., 2017; Wang H. et al., 2020). Moreover, the large specific surface area of 2D nanocrystals could expose more active sites, which could provide a large parameter space for the establishment of surficial modulations. The organization of surficial vacancies, single-atom modification, and dual-site components (as illustrated in Figure 1) are effective strategies for surficial modulation for enhanced photocatalytic performance, such as enlarging the light absorption region, facilitating electron–hole separation and transportation, and improving gas adsorption and activation properties. In this mini-review, the latest progress of the surficial modulations on 2D semiconductor photocatalysts with emphasis on their enhanced photocatalytic performance will be introduced.

## Surficial Vacancies

Normally, high-crystalline nanocrystals with a suitable band gap could feature a high build-in electronic field for a remarkable density of photo-induced charge carriers. By further modulating their surficial active sites, the light absorption and photo-induced charge transportation and separation processes could be further improved for enhanced photocatalytic performance (Sun et al., 2020). Two-dimensional semiconductor photocatalysts with atomically surficial modulation for selective photocatalytic reactions are introduced in Table 1. The surface modulation methods, active sites, photocatalyst conditions, and selectivity of the 2D photocatalysts described in the mini-review are highlighted. Among other surficial modulation strategies, the surficial vacancies (Figure 1A) could significantly change the local chemical coordination and surficial valence of the catalyst, which could not only mediate the surficial band gap but also enhance the activation of reactive molecules and charge transfer. Vacancies and adjustment of the surface band gap had a synergistic effect to reduce the recombination rate of photo-generated electrons and holes (Jo et al., 2019; Kong et al., 2020; Aggarwal et al., 2021). For example, the Chai group synthesized atomic-layered g-C_3_N_4_ with nitrogen vacancies (V_N_-g-C_3_N_4_), which exhibited high performance in converting CO_2_ to CH_4_ with high selectivity (Tang et al., 2019). The π-conjugated aromatic ring structure of g-C_3_N_4_ was distorted after the introduction of V_N_. At the same time, the introduced vacancy energy level changed the band gap. With increasing V_N_ density, the catalyst exhibited increased visible light absorption and enhanced reduction ability. In addition, the introduced V_N_ could suppress the recombination of electron–hole pairs because the vacancy level could act as a reservoir for trapping electrons. Therefore, the CH_4_ yield was 3.16 times and 5.14 times higher than its pristine ultrathin material and bulk material, respectively. The Hu group reported the preparation of 2D-TiO_2_ nanosheets having adjustable oxygen vacancies (V_O_) for selectively photocatalytic amine oxidation (Chen et al., 2020). They reported that the establishment of a hybrid energy level under the conduction band generated by surficial vacancies could decrease the band gaps, thereby enhancing the visible light catalytic activity. Meanwhile, the surficial oxygen vacancies facilitated the enhanced activation of O_2_ to ^1^O_2_ and O_2_^•−^. During the selectively light-driven catalytic process of benzylamine, O_2_ was more inclined to be adsorbed on the surficial V_O_ of TiO_2_, the photo-induced electrons could be transferred to the positively charged V_O_ reducing O_2_ to O_2_^•−^. The generated holes were largely transferred to the surface and oxidized most of the O_2_^•−^ to ^1^O_2_. Therefore, the generated ^1^O_2_ and O_2_^•−^ still participated in the oxidation of benzylamine simultaneously, showing selective and efficient conversion of benzylamine to imine.

**TABLE 1:** Comparison of 2D semiconductor photocatalysts with atomically surficial modulation for selective photocatalytic reactions.

Light source	System	Catalytic site	Photocatalytic reaction	Selectivity (%)	References
λ > 420 nm	V_O_ ^a^-TiO_2_ nanosheets	V_O_s and acidic sites	Amine oxidation	85.7	Chen et al. (2020)
AM 1.5	ZrS_1-y_S_2-x_ nanobelts	VS^2−^ ^b^ and VS_2_^2−^ ^c^	Benzylamine oxidation	>99	Tian et al. (2021)
λ > 420 nm	V_S_ ^d^-β-In_2_S_3_ nanosheets	V_S_s	Alcohol oxidation	>98	Sun et al. (2019)
Full-spectrum	V_Bi_ ^e^-BiOBr nanosheets	V_Bi_s	CO_2_ reduction	98	Di et al. (2019b)
Full-spectrum	Au/BP nanosheets	Au single atom	Methane oxidation	>99	Luo et al. (2021)
λ > 420 nm	Cu-C_3_N_4_ nanosheets	Cu-N_x_	Benzene oxidation	99.9	Xiao et al. (2020)
λ > 420 nm	CuIn_5_S_8_ nanosheets	Cu and In	CO_2_ reduction	−100	Li et al. (2019b)
AM 1.5	Pd-Ag-C_3_N_4_ nanosheets	Pd-Ag	CO_2_ reduction	-	Wang et al. (2020b)
AM 1.5	V_Zn_ ^f^-ZnIn_2_S_4_ atomic layers	V_Zn_s	CO_2_ reduction	-	Jiao et al. (2017)
Full-spectrum	Co-Bi_3_O_4_Br atomic layers	Co single atoms	CO_2_ reduction	-	Di et al. (2019a)
λ > 420 nm	Co-graphene nanosheets	Co single atoms	CO_2_ reduction	79.4	Gao et al. (2018)
λ > 400 nm	V_N_ ^g^-g-C_3_N_4_ nanosheets	V_N_s	CO_2_ reduction	-	Tang et al. (2019)
Full-spectrum	Pd-Ag-C_3_N_4_ nanosheets	Pd-Ag	Hydrogen production from water	-	Gallo et al. (2012)
λ > 420 nm	Ag-ZnIn_2_S_4_ monolayers	Ag single atoms and nanoholes	Overall water splitting	-	Pan et al. (2021)

a V_O_: oxygen vacancies.

b VS^2−^: sulfide anion vacancies.

c VS_2_^2−^: disulfide vacancies.

d V_S_: sulfur vacancies.

e V_Bi_: bismuth vacancies.

f V_Zn_: zinc vacancies.

g V_N_: nitrogen vacancies.

Another representative study by the Chen and Han group reported that ZrS_3_ nanoribbons (NBs) containing disulfide (S_2_^2−^) vacancies and sulfide anion (S^2−^) vacancies as a photocatalyst exhibited good photocatalytic performance for the formation of H_2_O_2_ and simultaneously selective conversion of benzylamine to benzonitrile, and the selectivity exceeded 99% (Tian et al., 2021). Similarly, they demonstrated that S_2_^2−^ vacancies in ZrS_3_ NBs accelerated the separation of photo-induced carriers, hole extraction, and kinetics of benzylamine oxidation. In another impressive study by the Xie group, cubic-phase In_2_S_3_ nanosheets were applied as a template, and S vacancies were introduced on their surface, which could enlarge the density of charge carriers involved in this reaction (Sun et al., 2019). It was indicated that the surficial S vacancies could promote the carrier separation and transfer and perform highly selective photocatalytic oxidation reactions by enhanced production of O_2_^•−^ as well. Theoretical simulations exhibited that the density of states of Valence Band Maximum (VBM) was significantly enhanced, which would inhibit electron–hole recombination.

Compared to the aforementioned anion vacancies, cation vacancies were relatively difficult to control, which could closely affect the conductivity and further affect the charge carrier mobility of the 2D nanocrystals in determining their separation of charge carriers (Hui et al., 2017). In addition, compared with the ideal vacancies, the metal vacancies on the engineering surface had an obvious electronic structure change due to the diversity of the electronic configuration and orbital of metal cations. The Sun and Xie group synthesized the one-unit-cell ZnIn_2_S_4_ layers with rich Zn vacancies (V_Zn_) to realize efficient and selective solar CO_2_ reduction performances (Jiao et al., 2017). The results of PL, SPV, and TA showed that the introduction of Zn vacancies into the ZnIn_2_S_4_ layer could significantly accelerate the charge transport, improve the electron–hole separation efficiency, and further increase the CO_2_ photoreduction efficiency. Additionally, DFT results of the density of states (DOS) indicated that the presence of zinc vacancy led to notable enhancement in the charge density of nearby sulfur atoms, which displayed that the electrons could be easily photoexcited to the conduction band and thus clustered around the zinc vacancies nearby the sulfur atoms and promoted enhanced carrier separation and transport. The Liu and Xia group synthesized BiOBr ultrathin nanosheets with Bi vacancies (V_Bi_-BiOBr) to optimize CO_2_ photoreduction performance (Di et al., 2019b). DFT calculations indicated that an increased charge density around the Fermi level due to the engineered Bi vacancies and V_Bi_-BiOBr could be endowed with increased charge carriers to participate in light-driven CO_2_ conversion with increased DOS. Therefore, the selectively photocatalytic CO yield of V_Bi_-BiOBr nanosheets in pure water was 20.1 μmol g^−1^ h^−1^, which was much higher than that of BiOBr nanosheets (5.3 μmol g^−1^ h^−1^).

## Single-Atom Modification

Compared to surficial vacancies, single-atom modification (Figure 1B) enabled by introducing guest atoms was another efficient strategy for surficial modulation of 2D semiconductor nanocrystals. The organized single-atom-modified photocatalysts could exhibit the advantages of single-atom catalysts, such as unsaturated coordination, a unique electronic structure, maximum atom utilization, and clear catalytic sites (Li et al., 2019a). Similar to surficial vacancies, the deposited guest atoms on semiconductor surfaces could act as active sites that could trap photo-induced charge carriers, thereby inhibiting charge carrier recombination and accelerating photocatalytic reaction (Wang et al., 2019). Hence, the single-atom modifications could be applied to regulate the dynamics of photogenerated charge transfer by shortening the charge migration distances and endowing strong interactions between metals and carriers (Gao et al., 2020). For example, the Zeng and Li group designed the single-atom Au-modified black phosphorus (Au_1_/BP) nanosheet catalyst for the selective oxidation process of methane to methanol (Luo et al., 2021). Under light irradiation, ^1^O_2_ generated by Au_1_/BP could react with water, forming adsorbed P-OH and P-OOH. P-OOH could be easily decomposed to generate •OH, while methane could react with P-OH to be dehydrated to form CH_3_• and be adsorbed on Au single atoms. Subsequently, •OH reacted with CH_3_• to generate CH_3_OH regardless of steric hindrance. In addition, Au single atoms stabilized CH_3_• against deeper dehydrogenation. Hence, Au_1_/BP can catalyze the partial oxidation of methane in an aqueous solution with selectivity >99%. The Liu, Song, and Xia group successfully incorporated Co single atoms into the Bi_3_O_4_Br atomic layer to construct a Co-Bi_3_O_4_Br catalyst (Di et al., 2019a). The introduced Co single atoms were beneficial to charge transfer, carrier separation, adsorption, and activation of CO_2_. Moreover, they could stabilize the COOH^•^ intermediate and modulate the rate-limiting step from COOH^•^ formation to CO^•^ desorption, which could lower the CO_2_ activation energy barrier. Utilizing single-atom Co and 2D ultrathin Bi_3_O_4_Br atomic layers, the photocatalyst could achieve a highly efficient photocatalytic CO_2_ conversion with a selective CO generation rate of 107.1 μmol g^−1^ h^−1^, which was approximately 4 and 32 times higher than that of atomic layer Bi_3_O_4_Br and bulk Bi_3_O_4_Br, respectively.

Meanwhile, the tunable surficial structure of the support materials could provide different coordination environments for guest atoms so that the geometric and electronic structures of these active sites would be tuned through the metal–support interaction, thereby changing the adsorption and strength of reactants, intermediates, or products (Gawande et al., 2020; Mitchell and Pérez-Ramírez, 2020). The Xiong group designed the partially oxidized graphene nanosheets (Co_1_-G) with single-atom Co sites and realized the highly selective photocatalytic conversion of CO_2_ to CO (Gao et al., 2018). The strong interaction between single-atom Co sites and supports could change the charge state of Co single atoms, thereby affecting the ability to activate CO_2_ molecules. Under the assistance of [Ru(bpy)_3_]Cl_2_, the turnover number (TON) for CO production reached a high value of 678 and the turnover frequency reached an unprecedented 3.77 min^−1^. TON was almost identical when the Co loading varied from 0.3 to 1.2 wt%, which indicated that each individual Co atom had been fully utilized as a photocatalytic site for CO_2_ reduction. The Fu, Jiang, and Zhang group reported the coordination of Cu atoms with N in C_3_N_4_ (Cu-N_x_) by thermal polymerization (Xiao et al., 2020). X-ray absorption spectroscopy combined with theoretical simulation displayed that each Cu atom could coordinate with three N atoms in one C_3_N_4_ layer or four N atoms in two adjacent C_3_N_4_ layers, forming two different types of Cu-N_x_ as efficient charge transport channels to promote rapid charge transfer. The catalyst exhibited an excellent visible light catalytic hydrogen evolution performance (212 mol h^−1^/0.02 g catalyst), which was 30 times higher than that of bulk C_3_N_4_. Additionally, the yield of benzene oxidation under visible light catalysis was 92.3%, and the selectivity reached 99.9%.

## Dual-Site Components

Compared to the aforementioned single-site modulation by vacancies and single atomic modification, surficial active sites coupling with other coordinated sites (Figure 1C), namely, dual-site component catalysts, were also reported by many groups, which generally exhibited higher selectivity because of the mediated work function, Fermi energy, and synergistic effects between the two components (Sankar et al., 2012). In this section, two main dual-site components were introduced, including metal–metal sites and metal–vacancy sites.

As for metal–metal sites, the engineered interatomic distances and reduced orbital overlap could induce the modulation in the band structure, affecting the density of states of the d-band and its position relative to the Fermi level (Rosseler et al., 2015). Therefore, the adsorption properties and reactivity of dual-metal component catalysts were different from those of single-atom modifications. Additionally, the dual-metal structure facilitates electron transfer between the two metal moieties, thereby further improving electron–hole separation (Ding et al., 2018). A representative study by the Sun and Xie group reported the synthesis of dual-metal site CuIn_5_S_8_ ultrathin nanosheets, which changed the intermediate configuration of the key reaction and adjusted the reaction barrier, thereby changing the reaction path and realizing the highly selective conversion of CO_2_ to CH_4_ (Li et al., 2019b). Both theoretical simulations and *in situ* infrared spectroscopy confirmed that the low-coordinated Cu and In sites could interact with CO_2_ molecules to form the extremely stable Cu-CO-In intermediate. Breaking the Cu-C bond and In-O bonds simultaneously needed to overcome a high reaction energy barrier to form free-state CO molecules. However, the reaction of hydrogenation on the C atom of this intermediate to form the CHO intermediate was exothermic and could proceed spontaneously. Photocatalytic experiments confirmed that the sulfur-defective CuIn_5_S_8_ ultrathin nanosheets could reduce CO_2_ to CH_4_ with close to 100% selectivity and an average yield of 8.7 μmol g^−1^ h^−1^ driven by visible light. Dual-metal alloy-modified photocatalysts could also exhibit improved catalytic performance (Tahir et al., 2017; Majeed et al., 2018), such as Pd-Au-modified g-C_3_N_4_ reported by the Feng group (Wang Z. et al., 2020). By regulating the proportion of Pd and Ag, the yields of CO and CH_4_ were adjusted, and selective conversion characteristics and high productivity could be achieved. The Santo group synthesized the dual-metal Au-Pt/TiO_2_ photocatalyst. Using ethanol as a sacrificial reagent, the obtained materials exhibited good hydrogen yields under both ultraviolet A and more sustainable simulated sunlight (Gallo et al., 2012). The interaction between Pt and Au led to a reduction in the strength of the metal–hydrogen bond, which means easier desorption of H_2_ from the metal surface and an enhanced electron capture capability of the materials.

Different from dual-metal components, single-atom modifications by introducing guest atoms could simultaneously generate surficial vacancies, forming metal-defect sites, which could further improve the performance and selectivity of photocatalysis due to the coupling effects. A recent study by the Zhang group reported that an engineered sulfide photocatalyst with surficial single atoms and vacancies exhibited a high-efficiency overall water splitting performance, which was obtained through a simple aqueous cation-exchange reaction between a 2D ZnIn_2_S_4_ monolayer and Ag^+^ (Pan et al., 2021). The vacancies in the Ag-ZnIn_2_S_4_ photocatalyst could act as the active centers, and the surficial isolated Ag atoms and their surrounding coordination environment could provide higher adsorption energy of H_2_O and OH^−^, promote the water oxidation process, and help to inhibit the light corrosion of the sulfide photocatalyst through holes. The dual-site synergistic effect served as the active site for overall water oxidation and reduction and realized extremely stable performance of photocatalytic overall water splitting without a co-catalyst. The Deskins group investigated the effects of surface oxygen vacancies and photoexcited electrons on the photocatalytic performance of TiO_2_-loaded Cu single atoms by DFT calculations and experiments (Chen et al., 2018). The single-atom Cu centers near the V_O_s had high selectivity for bent CO_2_ formation, which was beneficial to CO_2_ activation. In addition, the electron-rich nature of the single-atom Cu centers near the V_O_s increased the adsorption energy of CO_2_ at these sites, thereby accelerating the dissociation of CO_2_. However, Cu single atoms supported on the vacancy-free TiO_2_ surface did not exhibit the above properties. The presence of photoexcited electrons significantly increased the reaction rate on the surface of the TiO_2_ photocatalyst with Cu single atoms. In the absence of photoexcited electrons, the Cu/TiO_2_ surface was essentially inert. These results demonstrated that Cu single atoms, surface V_O_s, and photoexcited electrons work together to achieve high-efficiency and high-selectivity CO_2_ reduction.

## Conclusion and Perspective

In this mini-review, three surficial modulation strategies, namely, surficial vacancies, single-atom modulation, and dual-site components, are introduced for 2D semiconductor nanocrystal photocatalysts. These surficial modulation strategies have a positive impact on tuning key steps of photocatalytic reactions, such as enlarging the light absorption region, promoting electron–hole separation and transport, and facilitating gas adsorption and activation processes.

However, there are still many challenges for surficial modulation strategies for 2D semiconductor nanocrystal photocatalysts: 1) the leading reason for the enhancement of photocatalytic performance of 2D nanocrystals after surficial modulation needs more systematic and accurate test methods. Modulation of nanocrystals will cause changes in the overall properties of nanocrystals, but how to demonstrate the dominant reason needs more accurate research. 2) The explorations of the active sites and intermediate products require more detailed *in situ* characterizations such as *in situ* infrared, X-ray photoelectron spectroscopy, surface-enhanced Raman spectroscopy, etc., to further investigate the mechanism of photocatalysis. 3) A high density of surficial active sites could bring high catalytic activity, but the high loading of single atoms or defects could inevitably lead to aggregate or decomposition. Hence, how to maintain the stability of the photocatalyst is another important scientific issue. 4) Taking advantage of the photogenerated free radicals during photocatalytic reactions, realizing catalytic reactions which are difficult to be realized by conventional industrial catalysis could be an important direction for the future.
